# Estimating the number of cases of acute gastrointestinal illness (AGI) associated with Canadian municipal drinking water systems

**DOI:** 10.1017/S0950268815002083

**Published:** 2015-11-13

**Authors:** H. M. MURPHY, M. K. THOMAS, D. T. MEDEIROS, S. McFADYEN, K. D. M. PINTAR

**Affiliations:** 1 Centre for Food-borne, Environmental and Zoonotic Infectious Diseases, Public Health Agency of Canada, Guelph, ON, Canada; 2 Water and Air Quality Bureau, Healthy Environments and Consumer Safety Branch Health Canada, Ottawa, ON, Canada

**Keywords:** Epidemiology, estimating disease prevalence, public health, water (quality)

## Abstract

The estimated burden of endemic acute gastrointestinal illness (AGI) annually in Canada is 20·5 million cases. Approximately 4 million of these cases are domestically acquired and foodborne, yet the proportion of waterborne cases is unknown. A number of randomized controlled trials have been completed to estimate the influence of tap water from municipal drinking water plants on the burden of AGI. In Canada, 83% of the population (28 521 761 people) consumes tap water from municipal drinking water plants serving >1000 people. The drinking water-related AGI burden associated with the consumption of water from these systems in Canada is unknown. The objective of this research was to estimate the number of AGI cases attributable to consumption of drinking water from large municipal water supplies in Canada, using data from four household drinking water intervention trials. Canadian municipal water treatment systems were ranked into four categories based on source water type and quality, population size served, and treatment capability and barriers. The water treatment plants studied in the four household drinking water intervention trials were also ranked according to the aforementioned criteria, and the Canadian treatment plants were then scored against these criteria to develop four AGI risk groups. The proportion of illnesses attributed to distribution system events *vs.* source water quality/treatment failures was also estimated, to inform the focus of future intervention efforts. It is estimated that 334 966 cases (90% probability interval 183 006-501 026) of AGI per year are associated with the consumption of tap water from municipal systems that serve >1000 people in Canada. This study provides a framework for estimating the burden of waterborne illness at a national level and identifying existing knowledge gaps for future research and surveillance efforts, in Canada and abroad.

## INTRODUCTION

Enteric illness is largely underreported, and existing Canadian national and provincial surveillance systems for enteric illness often do not discriminate between infections caused by food, animal contact, person-to-person, environmental, or drinking water transmission, particularly for sporadic cases. Little is known regarding the magnitude and sources of waterborne illness in Canada.

In the 1990s, two non-blinded household water intervention trials were conducted in Laval, QC, a community on a surface water source (Canada) [1, 2]. These randomized controlled trials (RCTs) estimated that 0·126 and 0·0388 AGI cases per person per year, respectively, were attributable to drinking water. RCTs performed in the United States and Australia suggest between 0 and 0·0145 AGI cases/person per year may be attributable to tap water consumption from municipal systems [3–5].

In 2001, Environment Canada published an estimate of the burden of waterborne illness in Canada (90 000 cases of illness and nine deaths) [6] by multiplying an estimate from the United States by 10% [7]. This crude approach did not address differences between the two countries or variation in risk by system size, source water, or level of treatment employed. In 2012, Vinson [8] developed a crude estimate that 2·7 billion dollars is lost annually in Canada due to waterborne disease (not AGI-specific) from recreational and drinking water exposures. Neither of these estimates was derived using a systematic, evidence-based approach.

The Public Health Agency of Canada (PHAC) estimates that there are 20·5 million AGI cases each year (0·63 cases/person per year) [9]. This study provides the most accurate estimate to date of the burden of bacterial, viral and protozoan pathogens, and unknown agents that cause AGI in Canada. Of the overall burden, the PHAC estimates that 4 million are domestically acquired foodborne cases [9]. The remaining cases are attributed to water, animal contact, and person-to-person transmission.

In Canada, 84% (29 million) of the population is supplied tap water from a municipal drinking water plant serving >1000 people. According to data from Statistics Canada, 73% (25 million) of Canadians rely on a municipal system on a surface water source, including mixed ground/surface water; 1% (0·4 million) are on a groundwater under the direct influence of surface water (GUDI) supply; and, the remaining 10% (3·3 million) of Canadians rely on a groundwater source [10, 11].

The objective of this analysis was to estimate the number of AGI cases attributable to the consumption of tap water from Canadian municipal systems, and to determine the contribution of distribution systems to this estimate. Previously published RCTs were used to estimate the burden of endemic AGI associated with the consumption of tap water from these systems, elaborating on the approaches by Messner *et al.* [12] and Colford *et al.* [13]. This study was performed in tandem with a study to estimate the number of AGI cases associated with private wells and small municipal systems (serving <1000 people) in Canada [14].

## METHODS

**Fig. 1.**
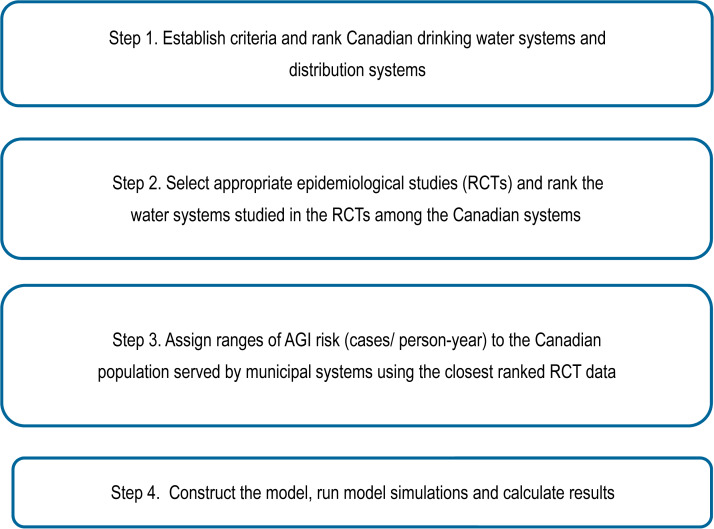
Key steps in the model development for the estimation of acute gastrointestinal illness (AGI) associated with the consumption of water from municipal water treatment systems serving >1000 people in Canada. RCT, Randomized controlled trial.

The methods used in this study were based on the US approach developed by Messner *et al.* [12], described below (Fig. 1).

### Step 1: Ranking of Canadian municipal water treatment systems serving >1000 people

Municipal water treatment systems were ranked using data from the 2011 Statistics Canada Survey of Drinking Water Plants [11]. This survey was administered to approximately 1800 drinking water treatment plants across Canada, each serving ⩾300 people. Drinking water treatment system characteristics, including source water type, population size served, treatment barriers in place, microbial raw water quality (based on indicator data) and treatment capability were used to rank systems. For each criterion, a series of scores was assigned. A higher ranking score equates to a lower public health risk. Each criterion and corresponding score is described below (Table 1). The criteria used in this analysis were reviewed by a Canadian drinking water treatment expert group.

**Table 1.** Canadian drinking water system ranking criteria and corresponding scores used for Canadian municipal systems serving >1000 people

Criteria	Subcategories	Score
Source water type	Surface water	1
	GUDI, mixed sources	2
	Groundwater	3
Microbial source water quality	Pristine (⩽0·1 c.f.u. *E. coli*/100 ml)	4
	Lightly impacted (>0·1 to ⩽10 c.f.u. *E. coli*/100 ml)	3
	Moderately impacted (>10 to ⩽100 c.f.u./100 ml)	2
	Highly impacted (>100 c.f.u./ 100 ml)	1
Log reduction capability of treatment system	See Table 2, minimum log reduction included based on treatment applied	0–6·42
Treatment barriers in place	See Table 2	0–4
Population served	>10 000	2
	1000–10 000	1

GUDI, Groundwater under the direct influence of surface water.

**Table 2.** Municipal water treatment system categories and estimated minimum log reductions by pathogen for each treatment category (Health Canada QMRA model) as well as corresponding minimum log reduction and barrier values assigned for the ranking of drinking water systems

Category	Treatment category details			Minimum (min) log_10_ reduction values/ranges for reference pathogens						
	Coagulation	Filtration	Disinfection*	*Campy*	*E. coli* O157	Rotavirus	*Crypto*	*Giardia*	Min. log reduction	Min. barriers
1	Coag/floc/sed	RG (coag/sed)	Optional chemical†	2·42	2·42	2·87	4·27	3·53	2·42	2
2	−	RG (direct filt)	Chemical†	2·37	1·49	0·62	2·97	2·87	0·62	2
3	−	RG (no coag)/SS or bag filt.	Chemical†	1·56	0·68	0·80	1·11	1·24	0·80	2
4	Coag/floc/sed	MF/UF and optional RG/SS	Chemical†	7·16	6·28	2·89	7·99	7·80	2·89	3
5	Coag/floc/sed	RG (coag/sed)	Chemical† and UV	8·43	8·05	6·42	8·70	7·54	6·42	4
6	−	RG (direct filt)	Chemical† and UV	7·37	6·99	4·7	7·40	6·87	4·70	3
7	−	RG (no coag)/SS or bag filt.	Chemical† and UV	6·56	6·18	4·88	5·54	5·24	4·88	3
8	Optional coag/floc/sed	MF/UF	Chemical† and UV	10·61	10·23	5·21	10·56	10·19	5·21	3
9	−	−	Cl_2_ or ClO_2_	4·00	4·00	4·01	0	0·21	0	1
10	−	−	Chloramine	1·01	0·13	0·03	0	0·01	0	1
11	−	−	Chemical† and UV	6·01	5·63	4·11	4·43	4·01	4·01	2
12	Coag/floc/sed	−	Chemical†	2·56	1·68	1·79	1·86	1·62	1·62	2
13	−	RG (no coag)/SS	−	0·55	0·55	0·77	1·11	1·23	0·55	1
14	No treatment			0	0	0	0	0	0	0
15	Optional coag/floc	MF/UF	Chemical†	5·61	4·73	1·13	6·13	6·19	1·13	2
16	−	RG (no coag)/SS and MF/UF	Chemical†	6·16	5·28	1·90	7·24	7·42	1·90	3
17	Coag/floc and/or sed/clar	−	Chemical† and UV	6·01	5·63	4·11	4·43	4·01	4·01	2
18	−	−	Chemical† (any two)	5·01	4·13	4·03	0	0·22	0	2
19	‘Other’ treatment		Treatment that did not fall into any of the above categories						2·32	1

*Campy, Campylobacter*; clar, clarification; coag, coagulation; *Crypto, Cryptosporidium*; filt, filtration; floc, flocculation; MF, microfiltration; QMRA, quantitative microbial risk assessment; RG, rapid granular filtration; sed, sedimentation; SS, slow sand filtration; UF, ultrafiltration.

* Assumes default values for disinfection contact time (20 min), concentration (0·20 mg/l), temperature (10 °C), pH (6·00), and UV dose (40 mJ/cm^2^) from Health Canada QMRA model (Health Canada [17]).

† For each log reduction value included, and for each pathogen listed, the value listed is the worst-case scenario, for any one of free chlorine, chloramine, ozone, or chlorine dioxide.

#### Source water type

Source water type was categorized into surface water, GUDI, mixed water sources, and groundwater. Surface water was assigned a score of 1, representing the greatest potential risk, from a microbial perspective, while GUDI and mixed sources were assigned a score of 2 [15, 16]. Groundwater was assigned a score of 3 as it is considered less microbially impacted than surface waters [15].

#### Microbial source water quality

Pathogen monitoring at most drinking water system intakes is not routinely performed in Canada – *E. coli* data serve as a proxy for faecal contamination and microbial quality. The microbial source water quality was estimated using reported average monthly maximum *E. coli* concentrations. These data were selected to represent the worst-case scenario source water quality and characterized using a previously described qualitative metric (pristine, lightly impacted, moderately impacted or heavily impacted) based on corresponding generic *E. coli* levels [17].

#### Reduction capability of the treatment system

Microbial reduction values on a logarithmic scale (log reduction) were assigned based on treatment type and performance (reduction or inactivation efficiency for five reference pathogens) to estimate drinking water treatment effectiveness. The minimum log reduction value achievable (as compiled in [16]) for either *Cryptosporidium, Giardia, Campylobacter, E. coli* and rotavirus was selected for each treatment component and summed for an overall log reduction. The municipal treatment plant data (Statistics Canada) were aggregated into groups of ⩾5 plants. Nineteen treatment categories were established and for each category, a minimum log reduction value was assigned (Table 2).

#### Number of treatment barriers

To account for the use of a multi-barrier approach, a criterion was established for the minimum number of treatment barriers in place (Table 1). A score of 0 was assigned for those plants with no treatment and a maximum score of 4 was assigned to those plants whose treatment included coagulation/flocculation/sedimentation, filtration, UV and chemical disinfection (defined as category 5 in Table 2).

#### Population served

The size of the population served was used as a proxy indicator for the level of operator training, the resources available to maintain and upgrade infrastructure and the overall robustness of the water quality monitoring system in place. In smaller systems, resources are limited [18] and operator training may also be limited [19]. Systems serving 1000–10 000 residents were assigned a score of 1, while systems serving ⩾10 000 residents were assigned a score of 2. Aggregation of plant survey data prevented the development of more narrow categories.

The population served by groundwater and surface water sources, categorized by dominant treatment type, is presented in Table 3.

**Table 3.** Distribution of Canadian population served by surface water and groundwater sources, classified by the dominant form of treatment (membrane filtration, media filtration, chemical disinfection, UV and no treatment)

Dominant form of treatment	Treatment categories*	Population served by surface water†	Population served by groundwater†
Membrane filtration	4, 15, 16	1 491 579	35 803
Media filtration	1, 2, 3, 13, 19	16 044 311	336 178
Chemical disinfection	9, 10, 12, 18	1 946 431	1 597 615
UV	5, 6, 7, 8, 11, 17	6 393 430	236 209
No treatment	14	16 450	423 758
Total		25 892 201	2 629 563

* Treatment categories correspond with those presented in Table 2.

† Population does not include Canadians that report consuming bottled water exclusively for bottled water consumers.

#### Ranking of Canadian distribution systems serving >1000 residents

The Canadian Infrastructure Report Card is a survey of municipal infrastructure that was conducted in 2009–2010 [20]. The survey addressed various aspects of municipal infrastructure, including drinking water. Seventy-five municipalities responded to the portion of the survey that addressed drinking water distribution systems, which included questions on system size, population served, system storage, pipe length, pipe material, and pipe condition state. Survey data and informal expert consultations were used to establish six criteria for distribution system ranking (Table 4).

**Table 4.** Canadian drinking water distribution system criteria and scores used in the ranking of Canadian distribution systems serving >1000 people

Criteria	Subcategories	Score
Pipe material	Metal only	1
	Metal and plastic	3
	Metal, plastic and other	5
	Metal and other	2
	Plastic only	7
	Plastic and other	6
	Other only	4
Pipe condition state*	Sum of very poor and poor, ⩾50%	1
	Sum of good and excellent, ⩾50%	3
	All other combinations	2
System size (pipe length)	Very large, >3000 km	1
	Large, 1001–3000 km	2
	Medium, 501–1000 km	3
	Small, <500 km	4
Population served	1000–5000	1
	>5000–10 000	2
	>10 000	3
Storage	Yes	1
	No	0
Chloramination	Yes	0
	No	1

* Categories based on Folkman [21] and the Canadian Infrastructure Report Card (Supplementary Table S1).

#### Pipe material

Pipe material used in distribution systems (metal, plastic and other) was available, but details on the proportion of each type used by each municipality were not provided. According to Folkman [21], metal pipes in Canada consist of ductile iron (23% of all pipe material), cast iron (18% of all pipe material) and some steel (1% of all pipe material). Plastic pipes are predominantly polyvinyl chloride (PVC) (43% of all pipe material), and ‘other’ pipe is a combination of asbestos cement (9% of all pipe material), concrete pressure pipe (4% of all pipe material) and other (2% of all pipe material). The published failure rates show that metal pipe (cast iron, ductile iron and steel, 35, 15·2 and 3·9 failures per 100 miles/year) is the most at risk of failing followed by other pipe materials (asbestos cement, concrete pressure pipe and other, 0·9–13·4 failures per 100 miles/year) and plastic pipe (PVC, 0·7 failures per 100 miles/year) [21]. This information was used to develop categories based on combinations of these pipe materials and their respective failure rates. For example, ‘plastic pipe only’ was assigned a score of 7 whereas ‘metal pipe only’ was assigned a score of 1 (Table 4).

#### Pipe condition

The Canadian Infrastructure Report Card asked utilities to report on the percentage of their pipe that was in very poor, poor, fair, good and excellent condition based on the definitions provided in Supplementary Table S1. These results were converted to scores using the classifications given in Table 4. For example, systems that reported a very poor or poor condition received a score of 1, while systems that reported good or excellent pipe condition were assigned a score of 3.

#### System size (pipe length)

System size, as defined by the length of pipe (in kilometers), was used as an indicator of risk. Due to their greater length of pipe runs, larger systems will face more hazards such as main breaks, dead ends, or older pipes (but not including weather events). For example, in Toronto, one of the largest cities in Canada, the average age of water pipes in 2002 was 90 years [22]. Scores from 1 to 4 were assigned to the systems using the following length categories: >3000 km (score = 1), 1001–3000 km (score = 2), 501–1000 km (score = 3), <500 km (score = 4) (see Table 4).

#### Population served

The number of people receiving water from each plant (population served) was used as a proxy indicator for the extent of operator training, resources available to maintain infrastructure and the overall robustness of the distribution system monitoring system in place. Small populations (1000–5000 people) were ranked as 1, medium populations (5001–10 000 people) were ranked as 2 and large populations (>10 000 people) were ranked as 3 (Table 4).

#### Storage

Storage in the distribution system was used as an indicator of the ability to maintain system pressure during power outages [23]. A score of 0 was assigned if storage was absent and a score of 1 was assigned if the municipality reported having storage (Table 4).

#### Chloramination

Although chloramine is an effective secondary disinfectant, due to its stability in the distribution system, it requires longer contact times than chlorine to kill potentially harmful microorganisms. Chloramination, in comparison to chlorination or chlorine dioxide, could increase the microbial risks associated with intrusion of contaminated water, given its association with biofilm growth and loss of residual [24, 25]. If chloramination was used, the plant was assigned a score of 0, *vs.* 1 for chlorine or chlorine dioxide use.

#### Weighting of criteria for treatment systems and distribution systems

The criteria outlined above were weighted, for a total score of 100 (Table 5).

**Table 5.** Weightings applied to the drinking water and distribution system criteria used in the ranking of Canadian water systems serving >1000 people

	Weight out of 100
Drinking water system criteria	
Source water type	10
Source water quality	30
Log reduction capability of the treatment system	30
Number of treatment barriers	20
Population served	10
Distribution system criteria	
Pipe material	20
Pipe condition	20
System size (pipe length)	15
Population served	15
Storage	15
Chloramination	15

### Step 2: Selection of appropriate epidemiological studies and subsequent ranking

#### Drinking water systems

Seven household drinking water trials (RCTs) were reviewed [26] (Table 6). Three studies were excluded – one [27] was a pilot study involving a small sample size; two focused on vulnerable populations (HIV-positive [28] and adults aged ⩾55 years [5]). The four remaining studies and the associated water treatment system rankings, based on the aforementioned criteria, were used (Supplementary Table S2).

**Table 6.** Summary of randomized controlled household drinking water intervention trials reviewed as potential data sources for estimating the burden of AGI associated with municipal drinking water systems

Author, publication date, ref.	Use of blinding/ use of an inactive (sham) treatment device	Study population	Study dates	Length of follow-up	Sample size (HH*/individuals)	HCGI incidence due to drinking water	Equivalent AGI* incidence due to drinking water
Payment *et al.* [1]	No	General population: homeowners with one child aged 2–12 years (did not exclude immunocompromised individuals)	Jan. 1988– June 1989	12 months	606/2408	0·26 cases/p-yr	0·126 cases/p-yr
Payment *et al.* [2]	No		Sept. 1993– Dec. 1994	16 months	1062/5253	0·08 cases/p-yr	0·0388 cases/p-yr
Hellard *et al.* [4]	Yes	General population: homeowners with one child aged 2–12 years (excluding those with immunocompromising conditions)	May 2000– May 2001	12 months	714/988	0·03 cases/p-yr	0·0145 cases/p-yr
Colford *et al.* [27]	Yes		Mar. 1999– Oct. 1999	4 months	77/236	0·85 cases/p-yr	0·412 cases/p-yr
Colford *et al.* [3]	Yes		Oct. 2000– May 2002	12 months	456/1296	−0·02 cases/p-yr	0 cases/p-yr
Colford *et al.* [28]	Yes	HIV + patients, aged >30 years, elderly aged >55 years (excluding immunocompromised individuals)	May 2000– May 2001	4 months	?/50	0·70 cases/p-yr	0·340 cases/p-yr
Colford *et al.* [5]	Yes		Apr. 2001– July 2006	12 months	714/988	0·265 cases/p-yr	0·128 cases/p-yr

AGI, Acute gastrointestinal illness; HCGI, highly credible gastrointestinal illness; HH, household; p-yr, person-years.

* The AGI rates published in the randomized controlled trials (RCTs) were documented in terms of HCGI which is a less restrictive definition of AGI. These HCGI rates were converted to AGI rates using estimated total Canadian HCGI and AGI rates of 1·3 cases/p-yr and 0·63 cases/p-yr, respectively (Thomas *et al*. [9]). Reported rates from the RCTs were multiplied by 0·63/1·3.

#### Distribution systems

Based on a systematic review of the literature [26], three studies were identified that examined the contribution of drinking water distribution systems to AGI [12, 29, 30] (Table 7). The Nygard *et al.* [30] and Lambertini *et al*. [29] studies were excluded as the systems studied were not representative of Canadian systems. Only the Messner *et al.* [12] study was used. Messner *et al.* [12] assumed that up to 50% of the difference in AGI observed between the first and second Payment *et al.* [1, 2] RCT studies could be attributable to the distribution system, which amounts to 0·02–0·06 cases of highly credible gastrointestinal illness (HCGI) per person per year. Given that the definition of HCGI is broader than that of AGI, and could include other causes of gastrointestinal illness, such as chronic illness, the HCGI estimates were adjusted, by multiplying by 0·63/1·3 (the proportion of AGI of the total HCGI estimated per person per year) [12]. Thus, 0·01–0·03 AGI cases per person per year were attributed to the distribution system and applied in this study to estimate the fraction of total AGI attributed to the distribution system [Supplementary Table S3. For example, in Supplementary Table S3, category 1, the percentage of AGI attributable to the distribution system ranged from 0 to 0·03/0·0388 (0–77%).] The Canadian distribution system studied by Payment *et al.* [1, 2] ranked at the 9·3 percentile in Canadian distribution systems.

**Table 7.** Summary of studies that examine AGI risk associated with drinking water distribution systems (DS)

Author, date, ref.	Location	Study description/results	Representative of Canada?	Selected	Percentile ranking among Canadian systems
Nygard *et al.* [30]	Norway	Study of AGI and DS events; up to 36% of AGI could be due to DS	Not representative: different pipe material and disinfection practices	No	n.a.
Lambertini *et al.* [29]	USA	Risk of DS in untreated groundwater supplies using QMRA; 1–4% of risk could be due to DS	May be representative of some small Canadian rural groundwater systems No residual maintained, therefore not representative of the majority of Canadian municipal systems.	No	n.a.
Messner *et al.* [12]	USA/Canada	USA burden estimate: two Payment studies [1, 2] used to estimate a proportion of AGI due to DS (range of 0·01–0·03 cases AGI/person-year)	Representative: Payment *et al.* studies [1, 2] were conducted in Laval, Canada	Yes	9·3

AGI, Acute gastrointestinal illness; QMRA, quantitative microbial risk assessment.

### Steps 3 and 4: Model building

Dividing Canadian municipal water treatment systems into four categories enabled delineation within each category with respect to treatment system(s) in place, source water and distribution system risk. The water treatment plants studied in the four RCTs were ranked according to the aforementioned criteria (Supplementary Table S2). The Canadian treatment plants were then scored against these criteria to develop four AGI risk groups (Supplementary Table S3). The corresponding Canadian population served and assigned risk of AGI (input in the model as a uniform distribution) are presented in Supplementary Table S3, and include an estimate of the role of the distribution system in the overall case estimates.

The AGI cases attributable to the consumption of municipal tap water were estimated (Supplementary Table S4) using Microsoft Excel Palisade @Risk software version 5.7 (Palisade Corp., USA). Latin hypercube simulations were run with 10 000 iterations and the mean, upper and lower 90% probability interval (PI) are presented (Table 8).

**Table 8.** Mean AGI cases attributable to municipal tap water consumption in Canada attributable to both source water/treatment and the distribution system, and corresponding AGI incidence rates

Category	Groupings of Canadian systems	Canadian population served by system categories (excluding bottled water)	Mean cases SW/TR (90% CI)	Mean cases DS (90% CI)	Mean total cases (90% CI)	Incidence rate (cases/p-yr)
1	Systems that are ⩽16·9 percentile SW/TR; ⩽9·3 percentile DS	1 069 447	30 745 (6436-69 114)	19 377 (1327-51 541)	50 121 (13 318-93 879)	0·047
2	Systems that are 17·0–42·9 percentile for SW/TR and ⩽9·3 percentile DS	2 000 635	16 138 (1181-40 621)	16 138 (1156-40 977)	32 278 (9869-57 331)	0·016
3	Systems that are 17·0–42·9 percentile for SW/TR and ⩾9·4 percentile DS	12 780 693	135 093 (35 495-277 142)	71 102 (4988-180 415)	206 195 (63 047-366 189)	0·016
4	Systems that are ⩾43percentile for SW/TR and ⩾9·4percentile DS	6 396 200	30 382 (9550-59 367)	15 991 (1267-38 210)	46 372 (17 541-75 185)	0·007
Total			212 358 (101 405-3-0 542)	122 608 (42 626-236 942)	334 966 (183 006-501 026)	0·015

AGI, Acute gastrointestinal illness; DS, distribution system; SW/TR, source water/treatment.

AGI attributable risk units = cases/person-year.

#### Model uncertainty

To capture the uncertainty associated with the estimates, model inputs were described, where possible, with parametric or non-parametric probability distributions (i.e. PERT or Uniform, depending on the data available). These capture the likely range and represent both uncertainty and variability in the data. A PERT distribution represents the minimum, maximum and most likely values for the value, whereas a Uniform distribution represents the minimum and maximum values for the value. For some aspects of the model, such as the method for ranking the water treatment plants in Canada, uncertainty could not be formally assessed because of the difficulty in determining how to quantify uncertainty and variability on a national scale.

## RESULTS

**Fig. 2.**
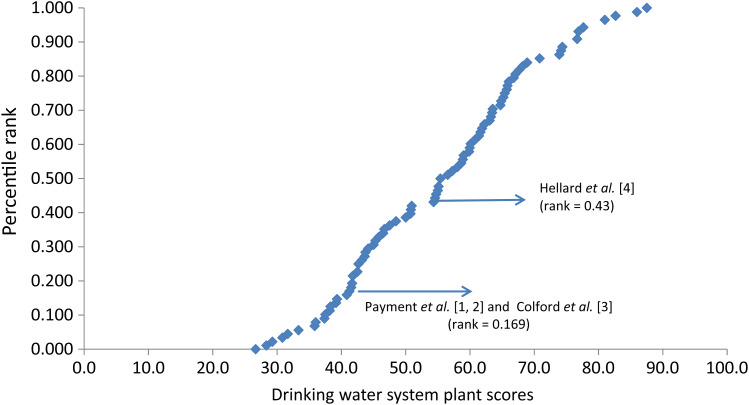
Ranking of Canadian municipal drinking water system plant scores including rankings of the systems studied in the randomized controlled trials.

**Fig. 3.**
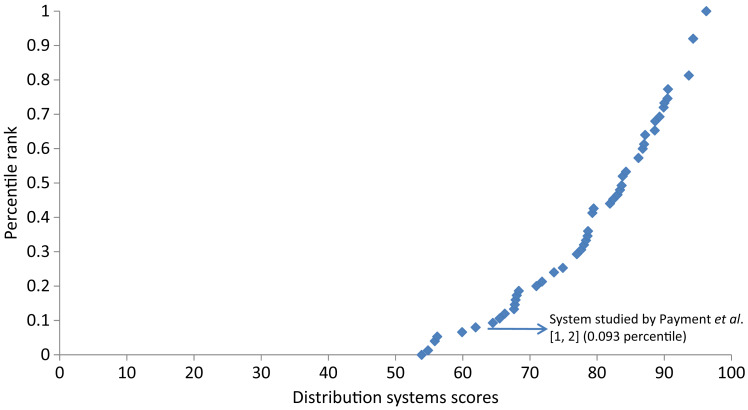
Ranking of 75 Canadian drinking water distribution system scores including ranking of the system studied by Payment *et al.* [1, 2].

Treatment plant scores ranged from 26·7 to 87·5, while the distribution system scores ranged from 53·9 to 96·3. Plotted distributions of these scores along with the rankings of the RCT systems [1–4] are presented in Figure 2. Figure 3 shows the curve of the distribution system scores and corresponding ranking of the Canadian distribution system [1, 2] that was used to calibrate the curve.

The Canadian drinking water systems were divided into four categories (Table 8) to facilitate ranking against the RCT results and the development of the model:

1. Systems ranking ⩽16·9 percentile for source water treatment and ⩽9·3 percentile for distribution systems (1·4 million people) – poorest quality.
2. Systems ranking between the 16·9 and 43·0 percentiles for source water/treatment and ⩽9·3 percentile for distribution systems (2 million people).
3. Systems ranking between the 16·9 and 43·0 percentiles for source water/treatment and >9·3 percentile for distribution systems (12·8 million people).
4. Systems that rank >43·0 percentile for source water/treatment and >9·3 percentile for distribution systems (6·4 million people) – best quality.

An estimated 334 966 (90% PI 183 066-501 026) AGI cases per year in Canada are attributed to the consumption of tap water from municipal systems serving >1000 people. Of these 334 966 AGI cases, an estimated 212 358 (90% PI 102 069-360 512) cases can be attributed to microbial source water quality and treatment; and 122 608 (90% PI 42 626-236 942) cases are attributed to distribution system events (Table 8).

An estimated 50 121 (15%) AGI cases are attributed to category 1 systems and 32 277 (9·6%) cases are associated with category 2 systems. The mean AGI incidence rate estimated for the population served by systems in category 1 is 0·047 cases/person-year. The mean incidence rate for the population served by category 1 and 2 systems (3 070 082) was 0·027 cases/person-year.

Category 2 systems serve approximately 2 million people, which we estimate fall between the 17th and 42·9th percentiles on the risk ranking curve (Fig. 3). The mean AGI incidence rate estimated for those served by category 2 systems is 0·016 cases/person-year, suggesting that these systems present less risk than the Payment *et al.* [1, 2] system, but a greater risk than the Hellard *et al.* [4] system.

The category 3 systems serve approximately 12·8 million people, of which nearly 11 million are on systems serving >10 000 people. The AGI incidence rate attributable to tap water for this category is low (0·016 cases/person-year), relative to rates estimated for US systems.

Systems ranking above the 43rd percentile fall into category 4 and serve 6·4 million Canadians, representing approximately 30% of all municipal systems serving >1000 people. The incidence rate for this category is 0·007 cases/person year (Table 8).

## DISCUSSION

This research is part of a comprehensive approach to attribute AGI in Canada to various sources. The objective of the work presented herein is to estimate the number of AGI cases attributable to the consumption of water from municipal systems serving >1000 people, and to apportion the number of cases related to the drinking water distribution and to the source water/treatment components of the system. The second objective of this work is to identify knowledge gaps and direct future research and surveillance efforts.

The estimated number of AGI cases attributable to municipal tap water consumption in Canada accounts for approximately 1·7% (334 966/20·5 million) of the overall burden of AGI in Canada [9].

The mean incidence rates estimated for each of the four categories of drinking water treatment systems are lower than those reported for community drinking water supplies in the United States (0·02–0·12 cases/person-year in the Messner *et al.* [12] study and 0·0156–0·043 cases/person-year in the Colford *et al.* [13] study). However, the range of uncertainty in these estimates overlaps, illustrating the difficulty in developing more precise estimates, given both the uncertainty and variability that exist in the current approach.

Category 1 systems represent ‘higher risk’ municipal systems, currently serving approximately 1·4 million people in Canada. Forty-six percent of systems in this category serve a population of >10 000 people, using a surface water source that is only treated by chlorine or chlorine dioxide (Table 3). The remaining 54% serve communities of 1000–10 000 people, generally considered to be small systems. Of these small systems, 38% rely on a surface water source, GUDI or a mixed water source with no treatment or only chemical disinfection, while 27% utilize a groundwater source with no treatment. Twenty percent are served by a moderate to highly impacted surface water source using only media filtration and chemical disinfection while the remaining 15% are served by various source waters with minimal amounts of treatment (1–2 barriers). The AGI incidence rates estimated for these systems (0·047 cases/person-year) are comparable to the upper range (for high-risk systems) reported by Colford *et al.* [13].

Most Canadians are supplied tap water from category 2 and 3 systems. Category 2 and 3 systems utilize a moderate to heavily impacted surface water source with conventional treatment. The AGI incidence rates attributable to tap water for these categories are moderate to low (0·027 and 0·016 cases/person-year) compared to the incidence rates estimated for similar drinking water systems in the United States [12, 13]. Category 4 systems rely on lightly impacted water sources with adequate treatment or impacted source waters with multiple barriers or treatment in place, including advanced treatment technologies (membrane filtration and/or UV disinfection). The incidence rate for this category is 0·007 cases/person-year, below the low range reported by Colford *et al.* [13] and nearly one tenth of the mean rate estimated by Messner *et al.* [12].

Based on the findings of this study, the most vulnerable municipal water treatment systems are in category 1. Over half are smaller systems serving between 1000 and 10 000 people, many of which have minimal barriers and rely predominately on chemical disinfection. The systems that pose the least risk are those equipped with advanced treatment systems with multiple barriers on relatively clean water sources. These findings are consistent with those of other researchers regarding potential risks to smaller systems; they also support the benefits of applying a multi-barrier approach to the delivery of drinking water [18, 31–33].

Over one third of all tap water-related AGI was attributed to the distribution system (122 608/334 966 = 36·6%). In distribution systems ranking ⩽9·3 percentile, 43% of all drinking water-related AGI was attributable to the distribution system, illustrating the susceptibility of these systems to distribution system intrusions. Nygard *et al.* [30] report that up to 36% of all AGI could be attributable to events in undisinfected distribution systems, while Lambertini *et al.* [29] suggest that 1–4% of tap water-related AGI could be due to the distribution system. In a recent meta-analysis, researchers reported that temporary water outages in distribution systems had a relative risk of gastrointestinal illness of 3·26, while chronic outages in intermittently operated systems had a relative risk of 1·61 [34]. These studies all demonstrate the influence that distribution systems have on downstream health risks. All distribution systems that ranked below the 9·3 percentile in this study use chloramination, known to promote biofilm growth, poor residual capacity, and most of these systems also reported no storage.

### Model and data limitations

Sensitivity analyses were performed by examining Spearman correlation coefficients (*r*_*s*_) to determine the impacts of model inputs on model predictions. The model was sensitive to: (1) the scoring/ranking of the systems in the RCTs with respect to Canadian systems; and (2) the estimated AGI rates reported from the household RCTs.

The ranking of Canadian systems was limited by the data available [11, 20]. Aggregating plants by treatment groupings will not capture the variation in individual plant performance and treatment barrier performance [33]. To estimate the number of AGI cases attributed to the distribution system, proportioning was based on survey data (plants self-reported the state of their distribution systems), introducing the possibility of reporting bias and the risk of underestimating the risks attributed to the distribution system. The use of *E. coli* indicator data as a proxy for source water microbial quality is not ideal. The presence of *E. coli* in water indicates faecal contamination, and thus, the strong potential for a health risk. However, its absence does not necessarily indicate that pathogens are also absent. Aggregated *E. coli* data (i.e. the mean of all monthly data from all aggregated plants by category) may not accurately reflect the concentrations of *E. coli* in source waters. Consequently, both pristine and heavily impacted source waters may have been missed. In addition, only two population groupings were possible (1000–10 000 and >10 000) due to data sharing restrictions from the surveys. The ranking approach would be strengthened if it were possible to evaluate plants individually, if pathogen monitoring data were available, and if plant-specific performance details and information about source water protection practices were available. In the absence of pathogen data, an index of source water quality such as the one presented by Hurley *et al.* [35], based on multiple water quality parameters, would be useful to characterize source water quality across the country (pH, turbidity, total organic carbon, *E. coli*, nitrate and nitrite as N, and temperature).

### AGI rates

The AGI rates applied in the current study were based on the results of four published RCTs, two of which were Canadian. These RCTs present the best epidemiological data available regarding illness attributable to tap water consumption. Ideally, this study would have been informed by a national intervention trial carried out in water supplies of varying sizes, on source waters of varying quality, and with varying treatment combinations. However, the costs of such a study are prohibitive. RCTs are expensive to conduct and the results are population and water system context-specific [36].

Although the RCTs were conducted using similar methodologies, the participants in the Payment *et al.* [1, 2] studies were not blinded. Non-blinded studies may bias results [37], although it is unknown whether the results would be an over- or under-representation of risk. This study could be strengthened if data were available from new RCTs conducted on a variety of source waters, including GUDI and groundwater sources, as well as different types of treatment systems.

## CONCLUSIONS

Based on model predictions, the consumption of municipal tap water from systems serving >1000 people may be responsible for an estimated 334 966 AGI cases annually (90% PI 183 066-501 026) in Canada, which accounts for roughly 1·7% of all AGI from all causes. Over one third of the cases may be attributable to the distribution system (122 608).

The results reinforce the understanding that Canadians served by smaller municipal systems (serving <10 000 residents) on surface water, GUDI or mixed water sources with no treatment or inadequate treatment (chemical disinfection only) are the most at risk of AGI [14]. An estimated 1·4 million people in Canada (4·1% of the population) are served by small water systems; 3% rely on groundwater and 1·5% rely on surface water, GUDI or mixed systems. Roughly half of Canadians receive drinking water from a municipal system that serves >10 000 residents, with moderately to heavily impacted surface waters and sufficient treatment (conventional treatment + chemical disinfection). This study demonstrates that the AGI risk to this population is very low (0·016 cases/person-year), and much lower than the risks estimated in comparable US systems (Messner *et al*. [12], ranging from 0·02 to 0·12 cases/person-year, while Colford *et al.* [13] estimated between 0·0156 and 0·043 cases/person-year). Water supplies that represent the lowest risk of AGI are those that rely on high-quality source water with at least one treatment barrier in place, as well as those systems that may have a more heavily impacted source water but rely on multiple barriers and advanced treatment such as membrane filtration and/or UV disinfection.

This study highlights the importance of implementing a multi-barrier approach for the delivery of safe drinking water. This starts with conducting a site-specific assessment that considers: (1) how variations in source water quality may contribute to microbiological risk and (2) the adequacy of existing treatment barriers (through the use of a variety of indicators – *E. coli,* turbidity, chlorine residual, etc.) in dealing with this risk. Assessments need to consider ‘worst case’ scenarios (e.g. hazardous events, failure of treatment barriers); and determine whether the quality of water is being maintained throughout the distribution system [31]. There are a number of strategies plants can then use to minimize risk, such as source water protection efforts, source water pathogen monitoring, implementing redundant treatment barriers, optimizing treatment, and maintenance of disinfectant residuals throughout the reach of the distribution system. However, these are situation-specific approaches, highlighting the need for a site-specific assessment of risk and the development of a water safety plan. A water safety plan ensures a system-wide approach to ensuring that the quality of water delivered to consumers is of good and consistent quality [38]. As a recent report highlights, a proactive approach to water management is a significant undertaking for any operator, municipality or regulator. One of the key indicators of success is community readiness. Those communities ready to prioritize water safety are more likely to have stakeholders aware of the need for change, have leaders who understand the issue, and have access to the resources needed to make the change possible [38].

Future estimates could benefit from:

1. Strengthening the treatment system ranking approach with plant-specific information regarding: plant performance (log reduction capabilities), source water pathogen occurrence, source water protection strategies, operator training, etc.
2. Future household water intervention trials on groundwater/GUDI sources and alternative treatment configurations.
3. Data on the state of Canadian drinking water distribution systems, including: frequency and causes of main breaks and distribution system events (e.g. low pressure events), residence times, pipe condition and proportion of pipe materials in individual systems.

## Supplementary material

For supplementary material accompanying this paper visit https://doi.org/10.1017/S0950268815002083. click here to view supplementary material
