# Supplementary material for: Estimating the number of cases of acute gastrointestinal illness (AGI) associated with Canadian municipal drinking water systems
Source: Epidemiol Infect. 2015 Nov 13;144(7):1371–85. doi: 10.1017/S0950268815002083 (PMC4823834; doi:10.1017/S0950268815002083)
Supplement: Supplementary file 1 [file S0950268815002083sup001.doc]

SUPPLEMENTARY MATERIAL

Table S1- Distribution system pipe condition categories as outlined in the Canadian Infrastructure Report Card (2013)

| **Physical Condition** | **Distribution System** |
| --- | --- |
| 5 – Excellent | - No structural defects. - Little or no water loss through leakage. |
| 4 – Good | - Minor cracking, spalling or signs of wear. - Deterioration causing minimal influences on levels of service - Less than 1 break/km/year. - Equivalent to OFWAT condition grade 2. |
| 3 – Fair | - Medium cracking, spalling or signs of wear. - Deterioration beginning to be reflected in deteriorating levels of service and/or increased operating costs. - Less than 3 breaks/km/year. - Equivalent to OFWAT condition grade 3. |
| 2 – Poor | - Fracture with deformation up to 10%. - Nearing the end of useful life, further deterioration likely, affecting levels of service. - Greater than or equal to 3-5 breaks/km/year. - Equivalent to OFWAT condition grade 4. |
| 1 – Very Poor | - Collapsed or collapse imminent. - No residual life expectancy, requires urgent replacement. - Equivalent to OFWAT condition grade 5. |

Table S2. Summary of selected RCTs, corresponding ranking scores and percentiles used to rank the systems among Canadian municipal drinking water systems

| **Author, publication date** | **Study area** | **Source water** | **Treatment** | **Treatment Category** | **Source water quality (cfu *E. coli*/ 100mL)** | **Population Served** | **Ranking Score** | **Percentile rank among Canadian systems** |
| --- | --- | --- | --- | --- | --- | --- | --- | --- |
| Payment  *et al.* (1991) | Laval, Canada | River water | Flocculation, Rapid sand filtration, ozonation, chemical disinfection | 1 | 367 cfu/ 100mL | >10,000 | 41.23 | 0.169 |
| Payment *et al.* (1997) | Laval, Canada | River water | Flocculation, Rapid sand filtration, ozonation, chemical disinfection | 1 | 367 cfu/ 100mL | >10,000 | 41.23 | 0.169 |
| Hellard *et al.* (2001) | Melbourne, Australia | Surface water source from protected catchment | Chlorination | 9 | 0 cfu/ 100mL | >10,000 | 53.33 | 0.430 |
| Colford *et al.* (2005) | Davenport, USA | River water | Conventional treatment with granular activated carbon/ sand filters & chlorination | 1 | Fecal coliforms  577 cfu/ 100mL | >10,000 | 41.23 | 0.169 |

^1^The AGI rates published in the various randomized controlled trials were documented in terms of highly credible gastrointestinal illness (HCGI) which is a less restrictive definition of AGI. These HCGI rates were converted to AGI rates using estimated total Canadian HCGI and AGI rates of 1.3 cases/ person-year and 0.63 cases/ person-year, respectively (Thomas *et al.* 2013). Reported rates from the RCTs were multiplied by 0.63/ 1.3.

Table S3. Summary of the categories resulting from the ranking of RCTs among Canadian drinking water systems and distribution systems including percentile ranking, population served, AGI incidence rates (cases/person-year) assigned for distribution system and source water/treatment risk

| **Category** | **Groupings of Canadian Systems** | **Percentile groupings for treatment system Ranking** | **Corresponding RCTs that fall in the category** | **Corresponding distribution system groupings, by percentile** | **Population Served** | **SW/TR AGI distribution assigned^4^** | **Distribution system AGI Risk^4^** | **Fraction of AGI due to Distribution^4^** |
| --- | --- | --- | --- | --- | --- | --- | --- | --- |
| 1 | Systems that are  ≤ 16.9%ile^1^ SW/TR^2^; ≤9.3%ile DS^3^ | 0 to 16.9 | Payment *et al.* 1991  Payment *et al.* 1997  Colford *et al.* 2005 | 0- 0.093  (13.8% population) | 1, 371,086 | Pert (0, 0.0388, 0.126)^1^ | 0.01-0.03 | Uniform  (0- 0.03/ 0.0388)  0- 77% |
| 2 | Systems that are 17.0-42.9%ile for SW/TR and  ≤9.3%ile DS | 17.0 to 42.9 | Assumption:  bordered by Payment *et al.* 1997 and Hellard *et al.* 2001 | 0- 0.093  (13.8% population) | 2,564,917 | Pert (0, 0.0145, 0.0388)^2^ | 0.01-0.03 | Uniform  (0- 0.03/ 0.0145)  0- 100% |
| 3 | Systems that are 17.0-42.9%ile for SW/TR and  ≥9.4%ile DS | 17.0 to 42.9 | Assmption:  bordered by Payment *et al.* 1997 and Hellard *et al.* 2001 | 0.094- 1.00  (86.2% population) | 16,385,504 | Pert (0, 0.0145, 0.0388)^2^ | 0-0.01 | Uniform (0- 0.01/ 0.0145)  0-69% |
| 4 | Systems that are ≥43%ile for SW/TR and  ≥9.4%ile DS | 43.0- 100 | Hellard *et al.* 2001 | 0.094-1.00  (86.2% population) | 8,200,256 | Pert (0, 0.00725,0.0145)^3^ | 0-0.01 | Uniform (0- 0.01/  0.0145)  0-69% |

^1^%ile= Percentile

^2^SW/TR = source water/treatment

^3^DS=distribution system

^4^AGI attributable risk units= cases/person-year

Table S4. Summary of calculations performed and key model inputs used in the determination of AGI attributable to municipal drinking water systems serving greater than 1000 people in Canada

| **Category** | **Total Population**  **(Column A)** | **Population (excluding bottled water)**  **(Column B= Column A-22%)** | **Fraction DS^1^**  **(Column C)** | **Fraction AGI SW/TR^2^**  **(Column D)** | **AGI Total**  **(Column E)^3^** | **AGI SW/TR**  **(Column F)** | **AGI DS**  **(Column G)** | **Cases SW/TR**  **(Column H)** | **Cases DS**  **(Column I)** | **Total Cases** |
| --- | --- | --- | --- | --- | --- | --- | --- | --- | --- | --- |
| 1 | 1,371,086 | 1,069,447 | 0-77% | 1- Column C | Pert( 0, 0.0388, 0.126) | D*E | **C*E** | B*F | B * G | Sum  (H +I) |
| 2 | 2,564,917 | 2,000,635 | 0-100% | 1- Column C | Pert (0, 0.0145, 0.0388) | D*E | **C*E** | B*F | B * G | Sum  (H+I) |
| 3 | 16,385,504 | 12,780,693 | 0-69% | 1- Column C | Pert (0, 0.0145, 0.0388) | D*E | **C*E** | B*F | B * G | Sum  (H+I) |
| 4 | 8,200,256 | 6,396,200 | 0-69% | 1- Column C | Pert(0, 0.00725,0.0145) | D*E | **C*E** | B*F | B * G | Sum  (H+I) |
| Total |  |  |  |  |  | = Sum Group 1-4 (Columns H+I) | | | | |

^1^DS- distribution system

^2^SW/TR – source water/ treatment

^3^AGI attributable risk units= cases/ person-year
